# Engineering 2D Silicene‐Based Mesoporous Nanomedicine for In Vivo Near‐Infrared‐Triggered Analgesia

**DOI:** 10.1002/advs.202202735

**Published:** 2022-06-24

**Authors:** Suqing Yin, Po Gao, Luodan Yu, Ling Zhu, Weifeng Yu, Yu Chen, Liqun Yang

**Affiliations:** ^1^ Department of Anesthesiology Renji Hospital Shanghai Jiao Tong University School of Medicine Shanghai 200127 P. R. China; ^2^ Materdicine Lab School of Life Sciences Shanghai University Shanghai 200444 P. R. China

**Keywords:** analgesia, nanomedicine, neuronal activation, photothermal excitation, ropivacaine

## Abstract

The utilization of local anesthetics for postoperative analgesia represents an effective approach, but generally suffers from short half‐lives and brachychronic local neurotoxicity. A desirable anesthetic with controllable and sustainable drug‐releasing performance for adequate analgesia effect is highly required. In this work, the core/shell‐structured two‐dimenional (2D) silicene nanosheets coated with mesoporous silica layer (abbreviated as Silicene@MSNs) have been rationally constructed as localized drug‐delivery system in sciatic nerve block to achieve on‐demand release of loaded ropivacaine (RP) in mesoporous silica layer for local analgesia. Based on the specific photothermal performance of 2D silicene core, this local anesthesia system can be triggered by near‐infrared laser to release the loaded RP, resulting in on‐demand and long‐lasting regional anesthesia. The analgesia effect is assessed by pain behavior tests, which demonstrates that the RP‐loaded Silicene@MSNs core/shell nanosystem behaves almost five times longer analgesia effect than free RP. Furthermore, the activation of pain‐related neurons in nerve conduction pathways is tested to explore the underlying analgesia mechanism, revealing that the designed nanosystem can improve the pain threshold, reduce the activation of neurons in dorsal root ganglion and excitability in spinal substantia gelatinosa neurons. This designed anesthetic nanomedicine provides a facile but effective methodology for long‐lasting regional anesthesia.

## Graphical abstract

A Silicene@MSNs local anesthetic nanomedicine with near‐infrared‐responsive drug‐releasing performance in the form of sciatic nerve block administration to effectively prolong the analgesia period by on‐demand release of loaded ropivacaine in mesoporous silica layer is developed. This work also verifies the analgesic effect by various neurobiological methods such as patch clamp, immunofluorescence, and pain behavior tests.

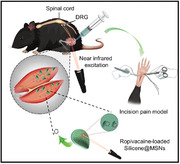

## 1 Introduction

Postoperative pain is an inevitable problem that associated with delayed recovery time, increased amount of opioid use, and higher health‐care costs under poor control. As one of the perioperative complications, postoperative pain bothers ≈50% of patients within 24 h after surgery,^[^ ^1^ ^]^ depending on the types of surgery clinically. The proportion of moderate pain occurrence ratio is about 40%, while the severe pain is 10–50%.^[^ ^2^ ^]^ Furthermore, a considerable part of the acute pain will transform into chronic pain. In this way, effective postoperative analgesia can bring huge economic benefits for improving the patient's quality of life, which is in line with the philosophy of enhanced recovery after surgery. Actually, even though the application of multiple analgesics, only about half of the patients received effective analgesia that partially depends on the type of surgery performed and analgesic/anesthetic intervention used.^[^ ^3^ ^]^

Local anesthesia is one way of postoperative pain management that can avoid the risk of opioid‐related adverse events. However, the short‐acting time of local anesthetics is usually within 2 or 4 h, which seriously restricts its application in postoperative analgesia. Ropivacaine (RP) is a relatively long‐acting local anesthetic with low cardiotoxicity, high clearance rate, and low neurotoxic, which is more suitable for nerve block compared to lidocaine and bupivacaine.^[^ ^4^ ^]^ However, multiple times of invasive manipulation is required to realize the long‐term analgesia, which may induce adverse local damage or potential neurotoxicity caused by local accumulation of analgesics. To address these critical issues, more drug carriers have been rationally built to realize controllable and sustainable drug release^[^ ^5^ ^]^ with the advancement of nanotechnology. Various forms of nanocarriers such as hollow mesoporous organosilica nanoparticles (HMONs),^[^ ^6^ ^]^ hydrogels,^[^ ^7^ ^]^ liposomes,^[^ ^8^ ^]^ polymeric formulation,^[^ ^9^, ^10^ ^]^ and metal nanoparticles^[^ ^11^ ^]^ have been designed, providing a promising strategy to optimize the pharmacokinetic profile of drugs without modifying their chemical structure. We have constructed a nanocarrier based on organic–inorganic hybrid HMONs‐based nanoplatforms.^[^ ^12^ ^]^ However, some of the studies need to optimize the controlled release of drugs, and most of them lack the evidence of nerve electrical activity inhibition during analgesia.^[^ ^13^ ^]^ Above all, it is still highly necessary to further explore the desirable anesthetics with controllable and sustainable drug‐releasing performance that can provide adequate analgesia effect for the utilization of local anesthetics generally suffering from short half‐lives and brachychronic local neurotoxicity.

**Scheme 1:**
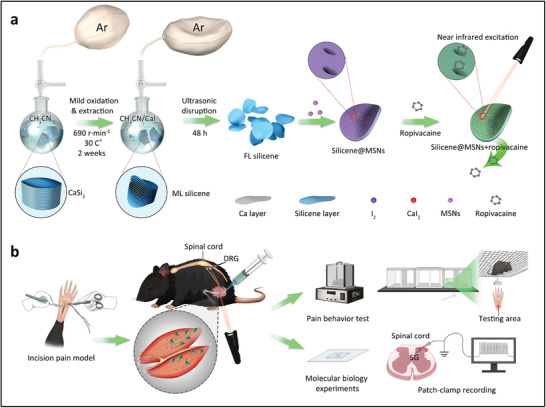
Schematic illustration of the preparation process of Silicene@MSNs loading with RP and their application as local anesthesia system. a) 2D silicene nanosheets originated from the bulk CaSi_2_. After coating with mesoporous silica layer, Silicene@MSNs loading with RP was constructed as local anesthetic nanomedicine. b) RP‐loaded Silicene@MSNs nanomedicine could be triggered by NIR laser to release the loaded RP, resulting in on‐demand and long‐lasting regional anesthesia.

Recently, 2D materials caused a flurry of nanotechnology for its unique physiochemical properties.^[^ ^14^ ^]^ Silicene, a 2D nanosheet with low‐buckled structure, has shown high potential in chemical sensing, bioimaging, drug delivery, and catalysis^[^ ^15^ ^]^ because of its intrinsic high biocompatibility, facile fabrication, and desirable photothermal‐conversion capability. Herein, in this work, we rationally designed a near‐infrared (NIR)‐responsive 2D silicene nanosheets coated with mesoporous silica layer (Silicene@MSNs) nanosheets as local anesthetic nanomedicine for on‐demand release of loaded RP (**Scheme** **1**), which was constructed via coating 2D silicene with mesoporous silica nanolayers (MSNs). On the one hand, the photothermal‐conversion property of silicene and the designed core/shell nanosheets were endowed with NIR‐responsive drug releasing performance and potential performances of thermotherapy for analgesia. On the other hand, high surface‐to‐volume ratio of silicene provides a desirable support matrix for mesoporous silica coating. To evaluate the analgesic effect of RP‐loaded Silicene@MSNs, plantar incision was selected as postoperative pain model with both the behavior tests and immunofluorescence staining of pain‐related neurons. It has been identified that the pain stimulus originated from the terminal of peripheral nerve fiber is transmitted to the posterior horn of the spinal cord via the dorsal root ganglion (DRG) and then transferred to a higher level of the central nervous system, eventually forming pain.^[^ ^16^ ^]^ During the formation of pain signal transmission, the corresponding neurons could be activated simultaneously. Furthermore, since the electrical activity of nerves is the lynchpin in the development of pain, we also applied the patch clamp to detect the pain‐related electrical activity of neurons in the substantia gelatinosa (SG) area of the spinal dorsal horn.

## 2 Results and Discussion

### 2.1 Synthesis and Characterization of 2D Core/Shell‐Structured Silicene@MSNs

**Figure 1:**
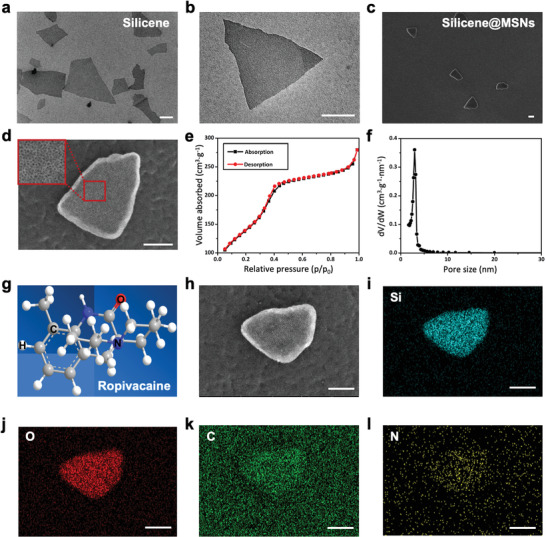
Structure/composition characterization of silicene, Silicene@MSNs and Silicene@MSNs loading with RP. TEM images of silicene at a) low and b) high magnifications. STEM images of Silicene@MSNs at c) low and d) high magnifications. The red box is a partially enlarged diagram of the mesoporous structure. e) N_2_ adsorption–desorption isotherm of 2D Silicene@MSNs. f) Corresponding pore‐size distribution of Silicene@MSNs. g) The chemical formula of RP. h) STEM and corresponding elemental mappings of i) Si, j) O, k) C, and l) N of Silicene@MSNs loading with RP. The underneath scale bar: 100 nm.

In this research, the NIR‐responsive 2D Silicene@MSNs nanosheets were constructed to load RP for controllable and long‐lasting analgesia (Scheme 1a). First, a typical wet‐chemical synthesis method was employed to fabricate free‐standing 2D silicene via a mild oxidation and delamination process.^[^ ^17^ ^]^ Herein, slightly overdose I_2_ was chosen to make sure the fully slow oxidation of CaSi_2_ precursor, thereby improving the yield and purity of 2D silicene. Transmission electron microscopy (TEM) image exhibited the almost electron‐transparent monolayer nanosheet structure of the fabricated 2D silicene under 48 h of further ultrasonic exfoliation (**Figure** **1**a,b). In order to endow the as‐synthesized 2D silicene nanosheets with drug‐loading capacity, a layer of MSNs in appropriate thickness was coated onto the surface of silicene, following the protocol described in the Experimental Section in detail. Dynamic light scattering (DLS) measurements illustrated that the average diameter was around 250 nm (Figure S1, Supporting Information). The structure of Silicene@MSNs was confirmed by the observation of scanning transmission electron microscopy (STEM) imaging (Figure 1c,d), of which the bright and dark field images are shown in Figure S2a–d in the Supporting Information.

### 2.2 Loading and Distribution of RP within Silicene@MSNs

To confirm the mesoporous structure of Silicene@MSNs, N_2_ absorption–desorption technique was performed. The Brunauer–Emmett–Teller (BET) surface areas of Silicene@MSNs and their corresponding pore volume were 506.1 m^2^ g^–1^ and 0.43 cm^3^ g^–1^, respectively, and the pore size was about 3.4 nm (Figure 1e,f). The large surface areas and the mesoporous nanostructure of Silicene@MSNs endowed the nanosheets with favorable drug‐loading performance.

RP is an aminoamide local anesthetic, which is mainly used for surgical area nerve block and epidural anesthesia.^[^ ^18^ ^]^ Like other local anesthetics, by blocking the flow of sodium ions into the nerve fiber cell membrane, it can reversibly impede the impulse conduction along the nerve fiber, which has the dual effects of anesthesia and analgesia.^[^ ^19^ ^]^ The molecular formula of RP is C_17_H_26_N_2_O, which is shown in Figure 1g. For the Silicene@MSNs nanocarrier, it only consists of Si and O elements (Silicene: Si; MSNs: Si/O). In this way, the C and N elements could be considered as characteristic distinguishing elements of RP that differ from the nanocarrier. STEM and elements mapping showed the increased distribution of C and N elements within Silicene@MSNs area, especially the N element (Figure 1h–l and Figure S2e–g, Supporting Information), indicating uniform loading of RP in nanoscale islands of Silicene@MSNs (Silicene@MSNs+RP).

### 2.3 NIR‐Activated Photothermal Effect of 2D Silicene@MSNs

**Figure 2:**
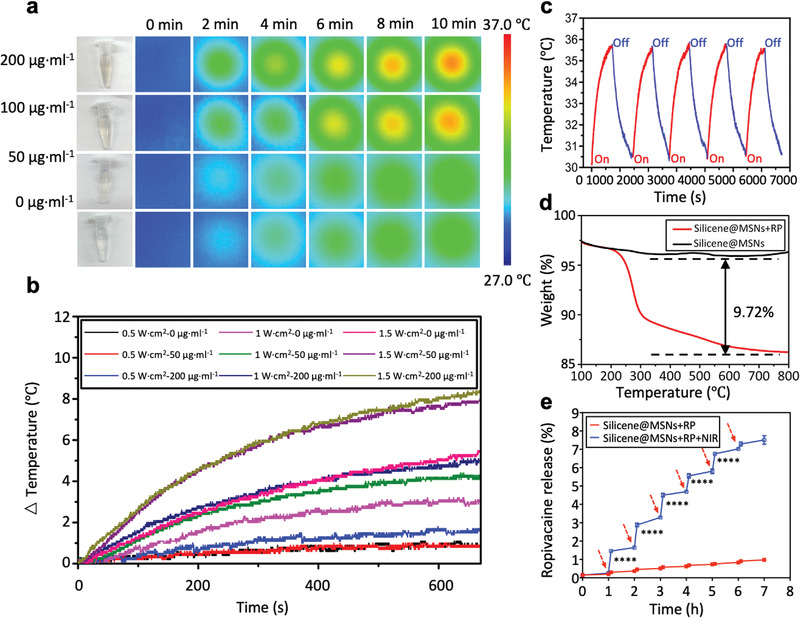
NIR‐activated photothermal property and loading capacity of Silicene@MSNs. a) Photothermal images of Silicene@MSNs in phosphate buffered saline solution at different concentrations and a series of time points. b) Photothermal‐heating curves of Silicene@MSNs NPs at different concentrations under different power of 1064 nm laser irradiation. c) Photothermal reaction of Silicene@MSNs exposed to 1064 nm laser irradiation in 1 W·cm^–2^ for 10 min and then the laser was turned off to cool down. d) Thermogravimetric analysis between Silicene@MSNs and Silicene@MSNs+RP. e) Release of RP with or without repeated 1064 nm NIR‐triggered irradiation, which was indicated by red arrows on the blue line (1 W·cm^–2^, 5 min). Data are medians ± SEM; ^****^ *p* < 0.0001.

The thermotherapy could relieve pain,^[^ ^20^ ^]^ and the IR may also induce auxiliary effect of analgesia via optimizing the nervous system^[^ ^21^ ^]^ and immune system.^[^ ^22^ ^]^ However, the thermotherapy for analgesia in previous studies was usually applied near or on the surface of the wound.^[^ ^20^, ^23^ ^]^ A previous study demonstrated that NIR low‐level laser therapy could reduce the plantar incision‐induced tactile allodynia when applied at the acupoint (ST36, Zusanli).^[^ ^24^ ^]^ Since the RP is a local anesthetic commonly used for nerve block, the expected potential performances of thermotherapy for analgesia around sciatic nerve trunk were explored in this study. The photothermal conversion ability of 2D silicene^[^ ^17^ ^]^ provides the possible thermotherapy effect of auxiliary analgesia while achieving controllable drug release. To ensure the penetration depth of excitation light, 1064 nm NIR was chosen as the excitation trigger. Furthermore, the IR thermal images were captured at different irradiation duration points, reflecting that the temperature rose in consonance with the increased NIR power (0.5/1/1.5 W·cm^–2^) and concentration of materials (0/50/100/200 µg·mL^–1^) in a nonlinear upward trend till the plateau period (**Figure** **2**a,b). Refer to the hot compress condition, it is generally regarded that around 42 ℃ is a more comfortable temperature. So, taking the base body temperature of 37 ℃ as a reference, the temperature rises about 5 ℃. Therefore, 1 W·cm^–2^ was selected as the more appropriate stimulation power density. The Silicene@MSNs at the concentration of 200 µg·mL^–1^ was exposed to 1064 nm NIR radiation under 1 W·cm^–2^ for 10 min and cooled down to initial temperature for five cycles (Figure 2c), indicating its high stability of photothermal property to be applied in this study.

In order to clarify the loading amount of RP in the nanocarrier, the Silicene@MSNs loaded with RP was freeze‐dried and subjected to thermogravimetric test, verifying that the loading amount was about 9.72% (Figure 2d). To further determine the NIR‐triggered releasing pattern of RP from Silicene@MSNs, the UV‐vis spectrophotometer was applied to measure the UV absorbance of RP according to the previously published method.^[^ ^6^ ^]^ Using phosphate buffer solution (PBS) as the solvent, RP features a characteristic absorption peak at 263 nm (Figure S3a, Supporting Information). According to the concentration of RP and the UV absorption peak at 263 nm, the calculated standard curve was shown as Figure S3b. To affirm the releasing efficiency of RP from Silicene@MSNs+RP, the releasing amount of RP in each of the two groups of Silicene@MSNs+RP with or without NIR irradiation was assessed (Figure 2e). Under the total six trigger cycles, much more RP was released in Silicene@MSNs+RP with NIR irradiation (Silicene@MSNs+RP+NIR) group, indicating well the NIR‐responsive and adjustable drug‐releasing performance of Silicene@MSNs‐based nanocarriers. Comparatively, in the Silicene@MSNs+RP group without NIR irradiation, only limited RP was released in each cycle.

### 2.4 Analgesic Effect of RP‐Loaded Silicene@MSNs Nanomedicine on Nociception

**Figure 3:**
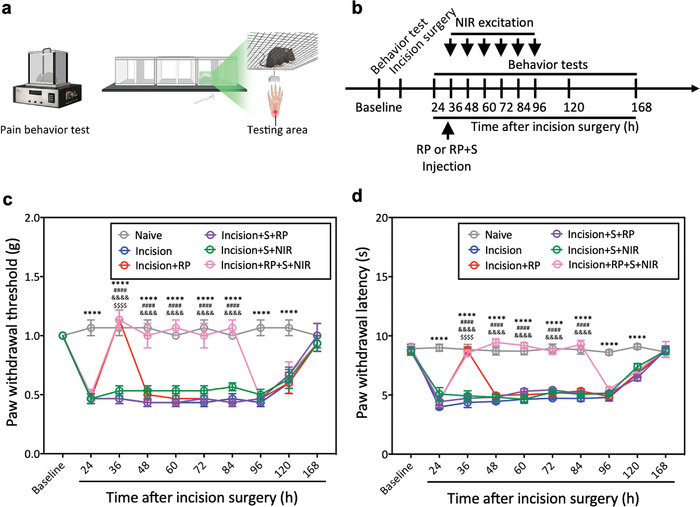
Representative time courses of pain assessments. a) Illustration of pain behavior tests: von Frey filaments and hot plate. b) Flow chart of behavioral test. c) Mechanical paw withdrawal threshold before and after treatments. c) Thermal paw withdrawal latency before and after treatments. Data are medians ± SEM; *n* = 6 per group. ^****^ *p* < 0.0001 between Naive and Incision groups, **^$$$$^** *p* < 0.0001 between Incision and Incision+RP groups, ^####^ *p* < 0.0001 between Incision+RP+S+NIR and Incision+S+NIR groups, ^&&&&^ *p* < 0.0001 between Incision+RP+S+NIR and Incision+RP+S groups, compared by using repeated‐measures ANOVA with Bonferroni's post hoc test.

To verify the analgesic effect of RP‐loaded Silicene@MSNs, plantar incision^[^ ^25^ ^]^ was selected as postoperative pain model and a single injection of RP‐loaded Silicene@MSNs around sciatic nerve was applied with NIR excitation for subsequent drug release (Scheme 1b). Behavior tests combined with various neurobiological methods, such as immunofluorescence and patch clamp further affirmed the satisfactory and long‐lasting analgesic effect of the nanomedicine. Both von Frey filaments and hot plate were applied to test the pain behavior (**Figure** **3**a) at different time points according to the flow chart (Figure 3b). Mechanical pain (Figure 3c) and thermal pain (Figure 3d) at different time points after the plantar incision surgery were tested to clarify the applicability of the as‐established analgesic nanosystem. According to the pre‐experimental results, the pain level of this model was relatively consistent about 1 day after operation. Therefore, in this experiment, we did not perform postoperative pain behavioral testing until 1 day after surgery to make sure the more stable, reliable, and comparable results.

Compared to mice without incision surgery (Naive group), the paw withdrawal threshold/latency of mice underwent plantar incision (Incision group) was obviously decreased until 7 days after incision surgery. A single administration of RP for sciatic nerve blocking at 34 h after incision showed a significant analgesic effect only at the nearest test time point (36 h after surgery) because the effective duration of RP is about 3–6 h,^[^ ^26^ ^]^ but no analgesic effect was monitored in mice underwent plantar incision with single injection of Silicene@MSNs loading RP around sciatic nerve without NIR excitation (Incision+RP+S group). However, after NIR excitation, mice underwent plantar incision with single injection of Silicene@MSNs loading RP around sciatic nerve combined with NIR excitation at each time point (Incision+RP+S+NIR group) brought out obvious analgesic effect after each excitation, which could withstand five excitations. To explore the expected potential performances of thermotherapy for analgesia around sciatic nerve trunk, we set a group of mice underwent plantar incision with single injection of Silicene@MSNs around sciatic nerve combined with NIR excitation at each time point (Incision+S+NIR group). As the application of NIR with Silicene@MSNs did not show obvious analgesic effect in behavior, the results indicated that the thermotherapy lacked the analgesia effect around sciatic nerve trunk. The negative result prompts researchers to search for other target of thermotherapy.

### 2.5 RP‐Loaded Silicene@MSNs Nanomedicine Inhibits the Activation of Pain‐Related Peripheral Neurons

**Figure 4:**
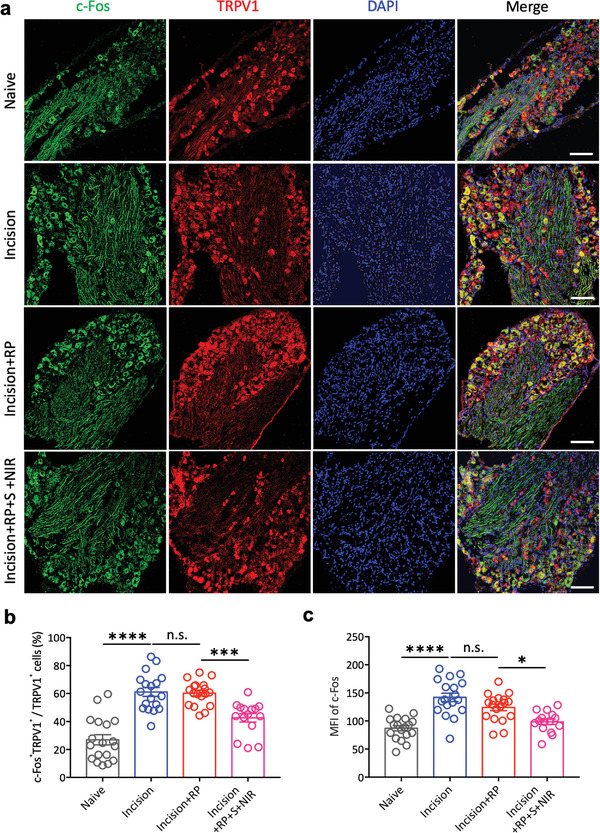
Immunofluorescence of c‐Fos and TRPV1 in L4, L5 DRG. a) Representative immunofluorescence of c‐Fos and TRPV1 in L4, L5 DRG 72 h after the plantar incision surgery. Green: c‐Fos^+^, Red: TRPV1^+^, Blue: DAPI^+^, Yellow: c‐Fos^+^TRPV1^+^. All scale bars = 100 µm. b) Statistical analysis of the proportion of co‐expression of c‐Fos and TRPV1 in TRPV1^+^ neurons in different groups (Naive, *n* = 18 slices from six mice; Incision, *n* = 18 slices from six mice; Incision+RP, *n* = 18 slices from six mice; Incision+RP+S+NIR, *n* = 15 slices from five mice; ***p < 0.001, ****p < 0.0001, n.s.: no statistically significant, one‐way ANOVA). c) Statistical analysis of MFI of c‐Fos in DRG in different groups (Naive, *n* = 18 slices from six mice; Incision, *n* = 18 slices from six mice; Incision+RP, *n* = 18 slices from six mice; Incision+RP+S+NIR, *n* = 15 slices from five mice; *p < 0.05, ****p < 0.0001, n.s.: no statistically significant, one‐way ANOVA).

In the pathway of pain signal, DRG and spinal dorsal horn are the key nodes of pain transmission.^[^ ^16^, ^27^ ^]^ Primary sensory neurons in DRG receive nociceptive stimuation, transmitting pain signals to the spinal dorsal horn and further to the higher central nervous system,^[^ ^28^ ^]^ which is activated and finally forms the pain sensations in the cortex.^[^ ^28^ ^]^ Therefore, we took the lumbosacral 4 (L4) and L5 DRGs and the spinal cord segment of the lumbosacral enlargement at 72 h after the incision surgery for immunofluorescence staining to observe and compare the activation of neurons. Transient receptor potential cation channel, subfamily V, member 1 (TRPV1) channel, is a key player in upward transmission of nociceptive sensations.^[^ ^29^ ^]^ TRPV1 and c‐Fos co‐staining of DRG neurons reflected the activation of TRPV1 positive neurons in DRG (**Figure** **4**a). Statistical analysis showed that both the proportion of c‐Fos positive neurons in TRPV1 neurons (Figure 4b) and mean fluorescence intensity (MFI) of c‐Fos (Figure 4c) were significantly increased in Incision group compared to Naive group. In Incision+RP+S+NIR group, both of them were significantly decreased compared to Incision group, while a single RP nerve block could not significantly amend the activation of c‐Fos. The results indicated that the treatment of Silicene@MSNs+RP with NIR excitation reduced the pain via suppressing the activation of pain‐related neurons in DRG.

**Figure 5:**
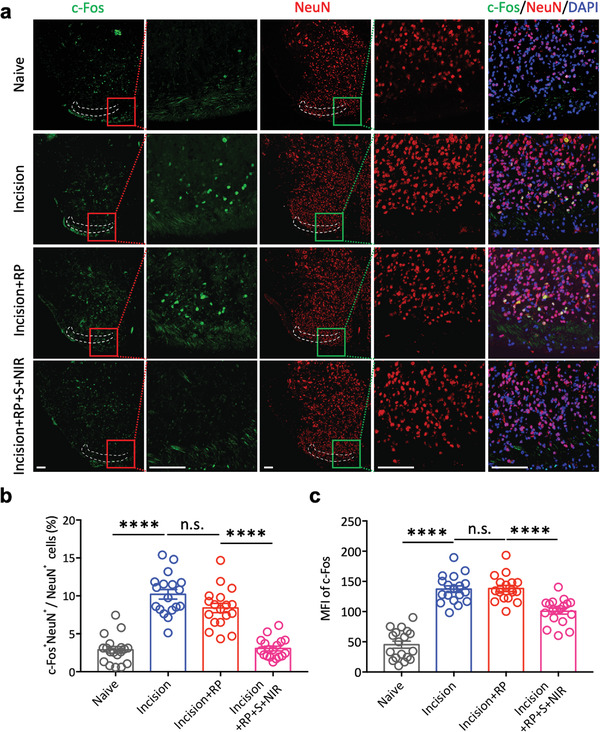
Immunofluorescence of c‐Fos and NeuN in SG area of spinal cord dorsal horn neurons. a) Representative immunofluorescence of c‐Fos and NeuN in SG area of spinal cord dorsal horn neurons 72 h after the plantar incision surgery. Green: c‐Fos^+^, Red: NeuN^+^, Blue: DAPI^+^, Yellow: c‐Fos^+^NeuN^+^. All scale bars = 100 µm. b) Statistical analysis of the proportion of co‐expression of c‐Fos and NeuN in NeuN^+^ neurons in different groups (*n* = 18 slices from six mice in each group; ****p < 0.0001, n.s.: no statistically significant, one‐way ANOVA). c) Statistical analysis of mean fluorescence intensity of c‐Fos and NeuN in SG area of spinal cord dorsal horn neurons in different groups (*n* = 18 slices from six mice in each group; ****p < 0.0001, n.s.: no statistically significant, one‐way ANOVA).

**Figure 6:**
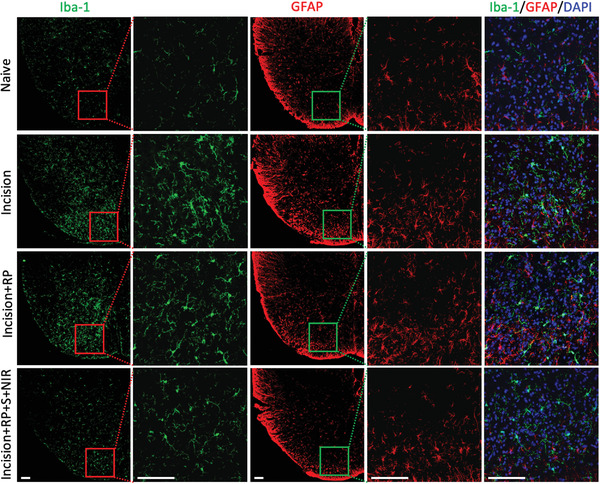
Immunofluorescence of Iba‐1 and GFAP in spinal cord dorsal horn. Green: Iba‐1^+^, Red: GFAP^+^, Blue: DAPI^+^. All scale bars = 100 µm.

Neurons were considered as the central role of information transmission in nervous system, as they can be excited for producing conductive action potentials. However, as another major component, glial cells had been authenticated for playing the role of protection, support, nutrition, and garbage removal in the development and maintenance of the nervous system. Actually, neuron‐glial cells signaling network in spinal dorsal horn participated in the process of mediating information transmission in the nervous system, especially in the transmission of noxious information.^[^ ^30^ ^]^ In the SG area of spinal dorsal horn, the Naive group had almost no activation of neurons, while in the Incision group, a large number of neurons were activated (**Figure** **5**a). Compared with the Incision group, the neuron activation in the mice underwent plantar incision with RP single injection around sciatic nerve at 1 day after incision (Incision+RP group) did not change significantly, while the c‐Fos positive cell ratio (Figure 5b) and the average fluorescence intensity of the neurons (Figure 5c) in the Incision+RP+S+NIR group were significantly reduced, which means that mice in this group received effective analgesia. Furthermore, it had been testified that the microglia and astrocytes in the L4‐5 segment of the spinal dorsal horn were obviously activated 3 days later in plantar incision pain model.^[^ ^31^ ^]^ The spinal cord was stained for ionized calcium binding adapter molecule 1 (Iba‐1) and glial fibrillary acidic protein (GFAP), which were used to label microglia and astrocytes, respectively (**Figure** **6**). Compared with the Naive group, microglia and astrocytes at spinal dorsal horn from the incision pain models were significantly activated. The single transient analgesic effect of RP could not inhibit the activation of microglia and astrocytes, while repeated intermittent analgesia could significantly inhibit the activation of pain‐related neurons, indicating Silicene@MSNs with RP activated by NIR could exert an effective and clear analgesic effect by inhibiting the activation of the ascending pain‐related neurons in the spinal cord.

### 2.6 RP‐Loaded Silicene@MSNs Nanomedicine Decreases Incision‐Induced Hyperexcitability in Spinal SG Neurons

Neuronal excitability is an important indicator of neuronal activity, reflecting the ability of neurons to generate action potentials. Previous studies have shown that the excitability of spinal SG neurons is increased in the state of pain, and the hyperexcitability of SG neurons mediates the occurrence and development of pain.^[^ ^32^ ^]^ RP is a long‐acting amide local analgesic, eliciting nerve block by reversibly inhibiting the sodium ion influx in nerve fibers.^[^ ^18^ ^]^ Therefore, whole‐cell patch‐clamp recordings were applied to measure the excitability of spinal SG neurons in different groups to reflect the production and conduction of pain. Spinal cord slices preparation was performed as described previously with minor modifications.^[^ ^33^ ^]^ Several indexes were used to evaluate the excitability of neurons, including resting membrane potential (RMP), threshold current required to induce action potential (AP), and the number of APs induced by the same depolarizing current. Electrophysiological experiments were carried out on the mice 3 days after operation. Acute SG slices were prepared from Naive mice, Incision mice, Incision+RP mice, and Incision+RP+S+NIR mice.

**Figure 7:**
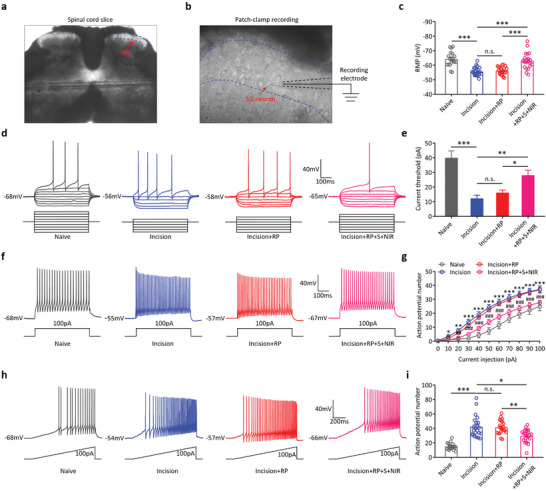
RP‐loaded Silicene@MSNs nanomedicine treatment decreases incision‐induced hyperexcitability in spinal SG neurons. a) Example of lumbar spinal cord slice. The red arrow indicates the area of SG. b) Example of targeted recording of an SG neuron. The red arrow indicates glass pipette targeting in an SG neuron. c) Statistical analysis of resting membrane potential (RMP) in different groups (Naive, *n* = 19 neurons from three mice; Incision, *n* = 22 neurons from four mice; Incision+RP, *n* = 21 neurons from four mice; Incision+RP+S+NIR, 21 neurons from four mice; ***p < 0.001, n.s.: no statistically significant, one‐way ANOVA). d) Representative traces of typical AP responses to a series of 500 ms hyper‐ and depolarizing current injections in 10 pA steps recorded from the SG neurons of Naive (gray), Incision (blue), Incision+RP (red), and Incision+RP+S+NIR (pink) mice, respectively. e) Statistical analysis of current threshold in different groups (Naive, *n* = 19 neurons from three mice; Incision, *n* = 22 neurons from four mice; Incision+RP, *n* = 21 neurons from four mice; Incision+RP+S+NIR, 21 neurons from four mice; *p < 0.05, **p < 0.01, ***p < 0.001, n.s.: no statistically significant, one‐way ANOVA). f) Representative traces of typical AP response to 100 pA depolarizing current injection recorded from the SG neurons of Naive (gray), Incision (blue), Incision+RP (red), and Incision+RP+S+NIR (pink) mice, respectively. g) Statistical analysis of AP number in different groups (Naive, *n* = 19 neurons from three mice; Incision, *n* = 22 neurons from four mice; Incision+RP, *n* = 21 neurons from four mice; Incision+RP+S+NIR, 21 neurons from four mice; *: Incision mice compared with Naive mice, ^#^: Incision+RP+S+NIR mice compared with Incision mice, *p < 0.05, **p < 0.01, ***p < 0.001, ^#^ *p* < 0.05, ^##^ *p* < 0.01, ^###^ *p* < 0.001, n.s.: no statistically significant). h) Representative APs of SG neurons from in response to a 100 pA ramp of depolarizing current over a 1000 ms stimulus. Representative APs from Naive (gray), Incision (blue), Incision+RP group mice (red), and Incision+RP+S+NIR (pink) mice, respectively. i) Statistical analysis of the APs induced by a 100 pA ramp of depolarizing current in different groups (Naive, *n* = 19 neurons from three mice; Incision, *n* = 22 neurons from four mice; Incision+RP, *n* = 21 neurons from four mice; Incision+RP+S+NIR, 21 neurons from four mice; *p < 0.05, **p < 0.01, ***p < 0.001, n.s.: no statistically significant, one‐way ANOVA).

The SG was discernible as a relatively translucent band across the dorsal horn, somewhat below the surface of the spinal cord (**Figure** **7**a). SG neurons were selected for the whole‐cell patch‐clamp recordings (Figure 7b). First, we found that the RMP of SG neurons in Incision mice was significantly higher compared to Naive mice (Incision −55.61 ± 0.64 mV vs Naive −64.28 ± 1.16 mV, *n* = 22, *n* = 19, respectively, *p* < 0.001), and RP+S+NIR treatment decreased the RMP of SG neurons in Incision mice (Incision+RP+S+NIR 63.06 ± 1.23 mV vs Incision −55.61 ± 0.64 mV, *n* = 21, *n* = 22, respectively, *p* < 0.001), but the RMP of SG neurons in Incision mice could not be changed by RP treatment (Figure 7c, Incision+RP 56.23 ± 0.59 mV vs Incision −55.61 ± 0.64 mV, *n* = 21, *n* = 22, respectively, *p* > 0.05). Next, we investigated the threshold currents required to elicit APs from SG neurons in the above four groups. The results showed that the threshold current was significantly lower in Incision mice compared to Naive mice (Figure 7d,e, Incision 12.27 ± 2.18 pA vs Naive 40.00 ± 4.71 pA, *n* = 22, *n* = 19, respectively, *p* < 0.001), and RP+S+NIR treatment increased the threshold current in Incision mice (Figure 7d,e, Incision+RP+S+NIR 28.10 ± 3.49 pA vs Incision 12.27 ± 2.18 pA, *n* = 21, *n* = 22, respectively, *p* < 0.01), while the threshold current in Incision mice could not be changed by RP treatment (Figure 7d,e, Incision+RP 16.19 ± 1.76 pA vs Incision 12.27 ± 2.18 pA, *n* = 21, *n* = 22, respectively, *p* > 0.05).

At the same time, the number of APs induced by steps current stimulation was also studied. We found that the number of APs evoked by the same current injection in the SG neurons of Incision mice was significantly higher than that in the Naive mice from the beginning of 10 pA current injection (Figure 7f,g, Incision *n* = 22, Naive *n* = 19, *p* < 0.05). RP+S+NIR treatment could reverse the increase in APs evoked by steps current stimulation (Figure 7f,g, Incision+RP+S+NIR *n* = 21, Incision *n* = 22, *p* < 0.05). However, RP treatment had no effect on the number of APs in incision mice (Figure 7f,g, Incision+RP *n* = 21, Incision *n* = 22, *p* > 0.05). In addition, we used a 100 pA depolarizing ramp of current injection to confirm the difference in AP. The results showed that the number of APs in Incision mice was significantly higher compared to Naive mice (Figure 7h,i, Incision 42.32 ± 3.14 vs Naive 14.95 ± 1.07, *n* = 22, *n* = 19, respectively, *p* < 0.001), and RP+S+NIR treatment could reverse the increase in APs (Figure 7h,i, Incision+RP+S+NIR 29.71 ± 1.95 vs Incision 42.32 ± 3.14, *n* = 21, *n* = 22, respectively, *p* < 0.05), while there was no difference in the number of APs between RP‐treated mice and Incision mice (Figure 7h,i, Incision+RP 41.43 ± 2.11 vs Incision 42.32 ± 3.14, *n* = 21, *n* = 22, respectively, *p* > 0.05). Together, these findings demonstrated that RP+S+NIR treatment could effectively inhibit the increase of excitability of spinal SG neurons in incision mice by decreasing the RMP, increasing the threshold current required to elicit APs, and reversing the increase of the AP number evoked by steps current stimulation or a 100 pA depolarizing ramp.

### 2.7 RP‐Loaded Silicene@MSNs Nanomedicine Treatment Reverses Incision‐Induced Increase in the Synaptic Transmission Efficiency of Spinal SG Neurons

**Figure 8:**
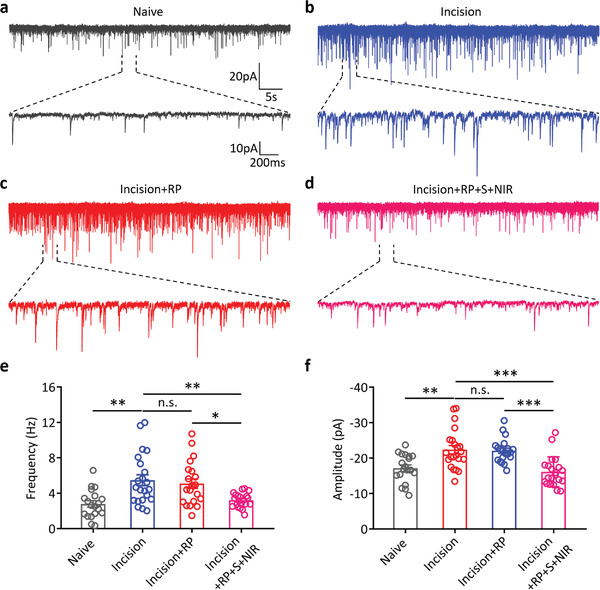
RP‐loaded Silicene@MSNs nanomedicine treatment reverses incision‐induced increase in sEPSCs of SG neurons. a–d) Representative traces showing sEPSCs of SG neurons from Naive (gray), Incision (blue), Incision+RP (red), and Incision+RP+S+NIR (pink) mice, respectively. e) Statistical analysis of the average frequency of sEPSCs in different groups (Naive, *n* = 19 neurons from three mice; Incision, *n* = 22 neurons from four mice; Incision+RP, *n* = 21 neurons from four mice; Incision+RP+S+NIR, 21 neurons from four mice; *p < 0.05, **p < 0.01, ***p < 0.001, n.s.: no statistically significant, one‐way ANOVA). f) Statistical analysis of the average amplitude of sEPSCs in different groups (Naive, *n* = 19 neurons from three mice; Incision, *n* = 22 neurons from four mice; Incision+RP, *n* = 21 neurons from four mice; Incision+RP+S+NIR, 21 neurons from four mice; **p < 0.01, ***p < 0.001, n.s.: no statistically significant, one‐way ANOVA).

The increase of neuronal excitability is often accompanied with the enhancement of excitatory synaptic transmission efficiency, which mediates the acceleration of pain signal transmission to higher central nervous system.^[^ ^34^ ^]^ Therefore, we further explored the effects of RP‐loaded Silicene@MSNs nanomedicine treatment on the spontaneous excitatory postsynaptic currents (sEPSCs) of spinal SG neurons in incision mice. The sEPSCs have two important indexes such as frequency and amplitude. Frequency represents the amount of neurotransmitters released from presynaptic membrane, and amplitude represents the function of receptors expressed on postsynaptic membrane. We herein found that both frequency and amplitude of sEPSCs of SG neurons in Incision mice were significantly higher compared to Naive mice (**Figure** **8**e,f, frequency: Incision 5.48 ± 0.61 Hz vs Naive 2.79 ± 0.36 Hz, *n* = 22, *n* = 19, respectively, *p* < 0.01; amplitude: −22.37 ± 1.18 pA vs Naive −17.15 ± 0.91 pA, *n* = 22, *n* = 19, respectively, *p* < 0.01), and RP+S+NIR treatment decreased both frequency and amplitude of sEPSCs of SG neurons in Incision mice (Figure 8e,f, frequency: Incision+RP+S+NIR 3.21 ± 0.17 Hz vs Incision 5.48 ± 0.61 Hz, *n* = 21, *n* = 22, respectively, *p* < 0.01; amplitude: Incision+RP+S+NIR −16.12 ± 0.92 pA vs Incision −22.37 ± 1.18 pA, *n* = 21, *n* = 22, respectively, *p* < 0.001), but both frequency and amplitude of sEPSCs of SG neurons in incision mice could not be changed by RP treatment (Figure 8e,f, frequency: Incision+RP 5.08 ± 0.53 Hz vs Incision 5.48 ± 0.61 Hz, *n* = 21, *n* = 22, respectively, *p* > 0.05; amplitude: Incision+RP −22.07 ± 0.72 pA vs Incision −22.37 ± 1.18 pA, *n* = 21, *n* = 22, respectively, *p* > 0.05). These results supported the conclusion that RP‐loaded Silicene@MSNs nanomedicine treatment could reduce the synaptic transmission efficiency via decreasing both frequency and amplitude of sEPSCs of spinal SG neurons in Incision mouse, thus achieving the goal of pain relief.

### 2.8 Toxicity Evaluation of RP‐Loaded Silicene@MSNs Nanomedicine

**Figure 9:**
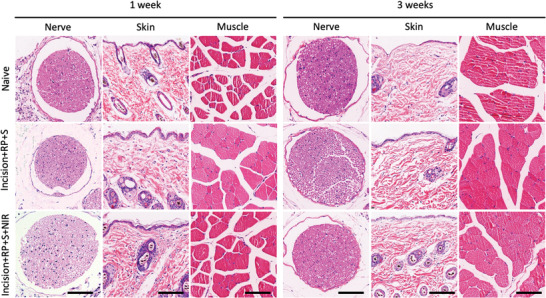
Biosafety assessment. Representative H&E‐stained sections of mice skin, muscle, and sciatic nerve to assess histocompatibility 1 week and 3 weeks in different groups. All scale bars = 100 µm.

To evaluate the biosafety of RP‐loaded Silicene@MSNs nanomedicine for in vivo applications, the single injection of RP‐loaded Silicene@MSNs around sciatic nerve combined with NIR excitation at each time point was conducted according to the flow chart in Figure 3b. 1 week and 3 weeks after the injection of nanomedicine, the mice were anaesthetized with isoflurane inhalation and perfused with 20 mL normal saline and 15 mL 4% paraformaldehyde sequentially. The skin, muscle, and nerve around the injection site were taken off for the further hematoxylin and eosin (H&E) staining histological analysis (**Figure** **9**). The results signified that there was no significant damage for sciatic nerve block of designed RP‐loaded Silicene@MSNs nanomedicine to the tissues surrounding the injection site, while the H&E results showed neither significant morphological change nor inflammatory cell infiltration in both short and long terms, indicating the high biosafety of RP‐loaded Silicene@MSNs administration.

## 3 Conclusions

In summary, this work successfully constructed a nanomedicine‐based delivery system (2D core/shell‐structured Silicene@MSNs) loading with RP triggered by NIR to achieve the controlled release of analgesic. In the plantar incision pain model, it substantially prolonged the pain relief duration in both animal behavioral tests and immunofluorescence assay so that the release of drugs could be spatially and temporally controlled, which was mastered by the whole‐cell patch‐clamp recording. In terms of in vivo biocompatibility, the designed 2D core/shell‐structured Silicene@MSNs nanomedicine features high biosafety, stability, and degradability.

## 4 Experimental Section

### Materials

Calcium silicide (CaSi_2_, 95%, Gelest), iodine (I_2_, 99.99%, Sigma‐Aldrich), acetonitrile (CH_3_CN, 99.8%, Sigma‐Aldrich), 1‐methyl‐2‐pyrrolidinone (NMP, 98%, Sigma‐Aldrich), double distilled water (Milli‐Q system, Millipore, USA), hexadecyltrimethylammonium chloride (CTAC, 99%, Adamas), triethylamine (TEA, 99%, Adamas), tetraethyl orthosilicate (TEOS, SiO_2_ iOrolidinGreagent), ethanol absolute (C_2_H_6_O, ≥ 99.7%, Shanghai Lingfeng Chemical Reagent Co. Ltd), hydrochloric acid (HCl, 36.0–38.0%, Shanghai Lingfeng Chemical Reagent Co. Ltd), and RP hydrochloride monohydrate (Adamas, 99%) were used. Antibodies which were used included Iba‐1 (WAKO, 019–19741), GFAP (Abcam, ab53554), TRPV1 (Abcam, ab5566), c‐Fos (CST, 2250S), Donkey Anti‐Rabbit IgG H&L (Abcam, ab150073), and Donkey Anti‐Goat IgG H&L (Abcam, ab150132). All chemicals used in slice preparation and electrophysiological recording were purchased from Sigma‐Aldrich.

### Synthesis of 2D Silicene

2D silicene was synthesized by a reported wet‐chemical exfoliation method. Typically, the accretion of I_2_ (6.35 g) was added into 750 mL acetonitrile in a round‐bottomed flask with precursor CaSi_2_ (2.4 g). After stirring 690 r min^–1^ at 30 ℃ for 2 weeks under the atmosphere of Ar, the sediment from centrifugation was washed with acetonitrile twice and with NMP once. The silicene nanosheets were sonicated in NMP for 48 h to break them into small pieces and then washed with double distilled water for three times.

### Characterization

The TEM images were acquired from a JEM‐2100F electron microscope (200 kV). STEM photographs and elements mapping were taken by a field‐emission Magellan 400 microscope. BET specific surface area was tested by automatic specific surface and porosity analyzer AUTOSORB IQ. DLS and zeta potential measurements were taken by Zetasizer Nanoseries. The vis–NIR absorption spectrum was recorded on UV‐3600 Shimadzu UV‐vis–NIR spectrometer. Thermal gravimetric analyzer TA was used to specify the carrier drug loading amount. NIR laser of 1064 nm was employed and the thermal‐field detection was collected by IR thermal camera.

### Synthesis of 2D Core/Shell‐Structured Silicene@MSNs

CTAC (10 g) and TEA (1 g) aqueous solution (TEA:ddH_2_O weight ratio = 1:10) was stirred under 80 ℃ water bath with 50 mL double distilled water. Pre‐prepared silicene (50 mg) was resuspended in double distilled water and added dropwise to the mixture above. Then, TEOS (320 µL) was added dropwise and reacted for 1 h. Finally, the precipitate after centrifugation was dispersed in 60 mL aqueous solution of hydrochloric acid and ethanol (ethanol:hydrochloric acid volume ratio = 9:1) and stirred under the 75 ℃ water bath for 6 h to extract redundant CTAC. The final production was washed with ethanol and double distilled water.

### Animal Experiments

Adult males 20 to 30 g C57BL/6 (Shanghai Jiesijie Laboratory Animal Co., Ltd.) were used in the experiments, of which the protocol were reviewed and approved by Ethic Committee of Shanghai University (ECSHU‐2021‐029). Mice were maintained under a 12 h–12 h light–dark photoperiod with free rodent diet and water.

### Pain Behavioral Testing

Calibrated von Frey filaments (VFFs) were used to reflect mechanical nociception by measuring withdrawal responses when stimulating the plantar surface of hind paw in mice while hot plate was chosen for hot pain behavior test. After baseline test, the mice with larger deviation were taken out and the remaining were divided into six groups (*n* = 6 of each group), including Naive‐operated mice (Naive group), control mice with plantar incision (Incision group), RP single injection around sciatic nerve at 1 day after incision (Incision+RP group), single injection of Silicene@MSNs loading RP around sciatic nerve after incision combined with NIR excitation at each time point (Incision+RP+S+NIR group), single injection of Silicene@MSNs loading RP around sciatic nerve after incision without NIR excitation (Incision+RP+S group), single injection of Silicene@MSNs around sciatic nerve after incision combined with NIR excitation at each time point (Incision+S+NIR group).

### Plantar Incision

Plantar incision was selected as postoperative pain model in this work. Under the inhalation anesthesia of isoflurane, the right hind paw was antiseptic prepared with iodophor and a 5 mm longitudinal incision through the skin and fascia of plantar foot was made from the edge of heel. The direction of incision was started 2 mm from heel and toward toes. Curved tweezer was used to separate the underlying muscle and 4‐0 suture was applied to close the incision. Finally, erythromycin ointment was applied on the surface of wound to prevent infection. Mice in Naive group underwent the consisted isoflurane anesthesia, antiseptic preparation, and antibiotics treatments without incision surgery.

### Response to von Frey Filaments

Individual mice were acclimated on pain test mesh rack covered with Plexiglas chambers for 30 min before the normal test. A quiet state and less voluntary activities were observed as well adapted. Calibrated von Frey filaments (0.008, 0.4, 0.6, 1, 1.4, 2 g bending force) were vertically thrust to the soles of mice adjacent to the incision until the filaments bent. Each monofilament was stimulated five times for 1 s with more than 1 min interval, starting at 0.008 g and ascending to 2 g gradually. Three or more times of obvious painful behavioral responses such as foot licking or foot withdrawal were considered as effective pain reaction. The previous gram was recorded as the mechanical pain threshold.

### Response to Hot Plate

Mice were placed on plate at room temperature to adapt for 30 min every day up to 3 days before the formal test. The hot plate test included placing mice on a hot plate (52.5 °C) and measuring the latency of hind paw‐licking, jumping, or rapid thumping of the hind paw. It was measured three times with an interval of 15 min and 30 s cut‐off time to prevent foot burns.

### Immunofluorescence

Mice were anesthetized with isoflurane inhalation and perfused with 20 mL normal saline and 15 mL 4% paraformaldehyde sequentially. The DRG of 4, 5 lumbar segments (L4, L5), and spinal cord segments of lumbosacral enlargements were dissected out. After postfix and sucrose gradient dehydration, DRG was sectioned frozen at 10 µm and spinal cords at 20 µm thickness. TRPV1 and c‐Fos staining in DRG reflected the activation of TRPV1 positive neurons, while in spinal cord NeuN and c‐Fos marked the activation of neurons, as well as Iba‐1 and GFAP were used to label microglia and astrocytes, respectively. The number of positive neurons and MFI were calculated by Image J.

### Preparation of Spinal Cord Slice

Briefly, adult (8–10 weeks old) mice were deeply anesthetized with overdose of pentobarbital sodium and decapitated. The lumbar spinal cord L4‐5 was quickly removed to ice‐cold oxygenated (95% O_2_ and 5% CO_2_) NMDG artificial cerebrospinal fluid (ACSF) (in × 10^−3^ m, 92 NMDG, 2.5 KCl, 1.25 NaH_2_PO_4_, 30 NaHCO_3_, 20 HEPES, 25 glucose, 2 thiourea, 5 sodium ascorbate, 3 sodium pyruvate, 0.5 CaCl_2_·2H_2_O, and 10 MgSO_4_·7H_2_O, pH were adjusted to 7.3 with concentrated HCl, 295–305 mOsm).

Transverse spinal cord slices (300 µm) were cut using a vibratome (VT1200S, Leica Microsystems, Germany) in the ice‐cold NMDG ACSF. The slices were incubated at 33 ℃ in oxygenated NMDG ACSF for 10 min. Then the slices were transferred to the holding chamber and incubated at room temperature in HEPES holding aCSF (in × 10^−3^ m, 92 NaCl, 2.5 KCl, 1.25 NaH_2_PO_4_, 30 NaHCO_3_, 20 HEPES, 25 glucose, 2 thiourea, 5 sodium ascorbate, 3 sodium pyruvate, 2 CaCl_2_·2H_2_O, and 2 MgSO_4_·7H_2_O) for at least 1 h. A single slice was then transferred into the recording chamber and perfused with oxygenated recording aCSF (in × 10^−3^ m, 125 NaCl, 2.5 KCl, 2 CaCl_2_, 1 MgCl_2_, 1.25 NaH_2_PO_4_, 25 NaHCO_3_, and 12.5 D‐glucose, pH 7.3–7.4) at a rate of 2 mL min^–1^.

### Whole‐Cell Patch‐Clamp Recording

The whole‐cell patch‐clamp recordings in current‐ and voltage‐clamp mode were made from SG neurons of spinal dorsal horn under differential interference contrast optics (BX51WI, Olympus, Tokyo, Japan), respectively. The recording pipettes (6–8 MΩ resistances) were pulled by a horizontal micropipette puller (P‐1000, Sutter Instruments) from borosilicate capillaries and filled with the following internal solution (in × 10^−3^ m: 120 K‐gluconate, 5 NaCl, 10 KCL, 1 CaCl_2_⋅2H_2_O, 2 MgCl_2_⋅6H_2_O, 11 EGTA, 10 HEPES, 2 Mg‐ATP, and 1 Li‐GTP, pH was adjusted to 7.3 with Tris‐base). A 5 min equilibration period was allowed to reach the steady state after whole cell access was established. The resting membrane potential was continuously recorded with *I* = 0 current‐clamp mode. To measure excitability of SG neurons, the action potential (AP) current threshold was tested using a series of 500 ms hyper‐ and depolarizing current injections in 10 pA steps ranging from −40 to 100 pA. The current that induced the first AP was defined as threshold current and the number of APs induced by each depolarizing current was recorded. In addition, the neurons were injected with a 100 pA depolarizing ramp of current (1 s in duration) and the number of resulting APs was recorded. The membrane potential was held at −70 mV for recording sEPSCs with voltage clamp mode. Membrane voltage and current were amplified using MultiClamp 700B (Molecular Devices, Sunnyvale, Calif, USA), filtered at 2 kHz and digitized at 10 kHz. Data were acquired using pClamp 10.7 software (Molecular Devices, Sunnyvale, Calif, USA). Only neurons with access resistance <20 MΩ and input resistance >100 MΩ were studied. Neurons were discarded if the access or input resistance changed by more than 20%.

### Statistical Analysis

Data is presented as the mean ± SEM. Statistical analysis was performed using GraphPad Prism7 (GraphPad Software Inc., USA). Unpaired two‐tailed Student's *t* test was used to examine the difference between two groups. Behavioral data were analyzed using two‐way repeated‐measures analysis of variance (ANOVA) followed by the Bonferroni's post hoc test. Multiple group comparison was performed with one‐way ANOVA analysis followed by the Tukey's post hoc test. Differences were considered statistically significant when a *p* value was less than 0.05.

## Conflict of Interest

The authors declare no conflict of interest.

## Supporting information

Supporting Information

## Data Availability

The data that support the findings of this study are available from the corresponding author upon reasonable request.
