# Supplementary material for: Engineering 2D Silicene‐Based Mesoporous Nanomedicine for In Vivo Near‐Infrared‐Triggered Analgesia
Source: Adv Sci (Weinh). 2022 Jun 24;9(25):2202735. doi: 10.1002/advs.202202735 (PMC9443434; doi:10.1002/advs.202202735)
Supplement: Supplementary file 1 — Supporting Information [file ADVS-9-2202735-s001.pdf]

Copyright WILEY-VCH Verlag GmbH & Co. KGaA, 69469 Weinheim, Germany, 2021.

Supporting Information

Engineering Two-Dimensional Silicene-Based Mesoporous Nanomedicine for In Vivo Near Infrared-Triggered Analgesia

Suqing Yin[#], Po Gao[#], Luodan Yu[#], Ling Zhu, Weifeng Yu*, Yu Chen* and Liqun Yang*

S. Yin, Dr. P Gao, Dr. L. Zhu, Prof. W. Yu, Prof. L. Yang

Department of Anesthesiology, Renji Hospital, Shanghai Jiao Tong University School of Medicine, Shanghai 200127, P. R. China.

E-mail: yangliqun@renji.com (L. Yang), yuweifeng@renji.com (W. Yu)

Dr. L. Yu, Prof. Y. Chen

Materdicine Lab, School of Life Sciences, Shanghai University, Shanghai 200444, P. R. China.

E-mail: chenyuedu@shu.edu.cn

# Equal contribution author.

Supplementary figures

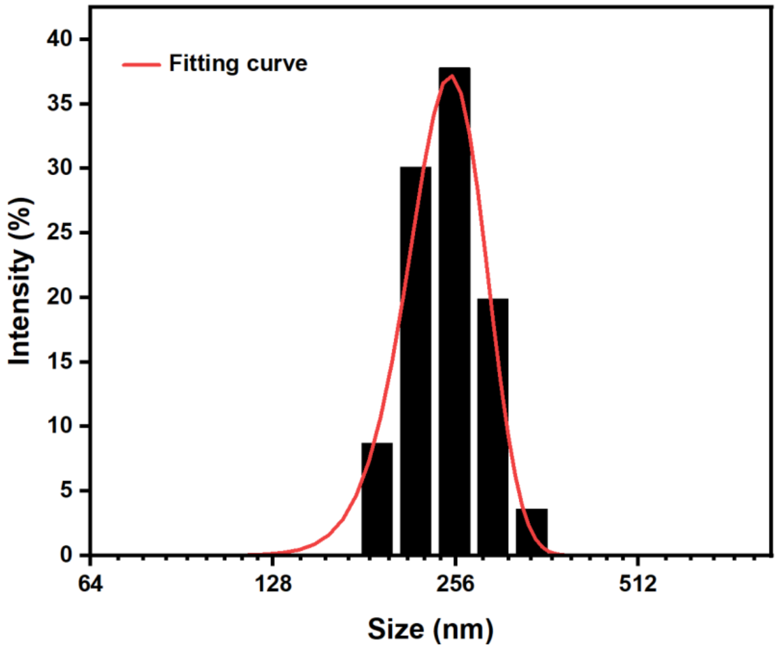

Figure S1. Hydrodynamic diameters of 2D Silicene@MSNs dispersed in phosphate buffer saline (PBS) analyzed by DLS.

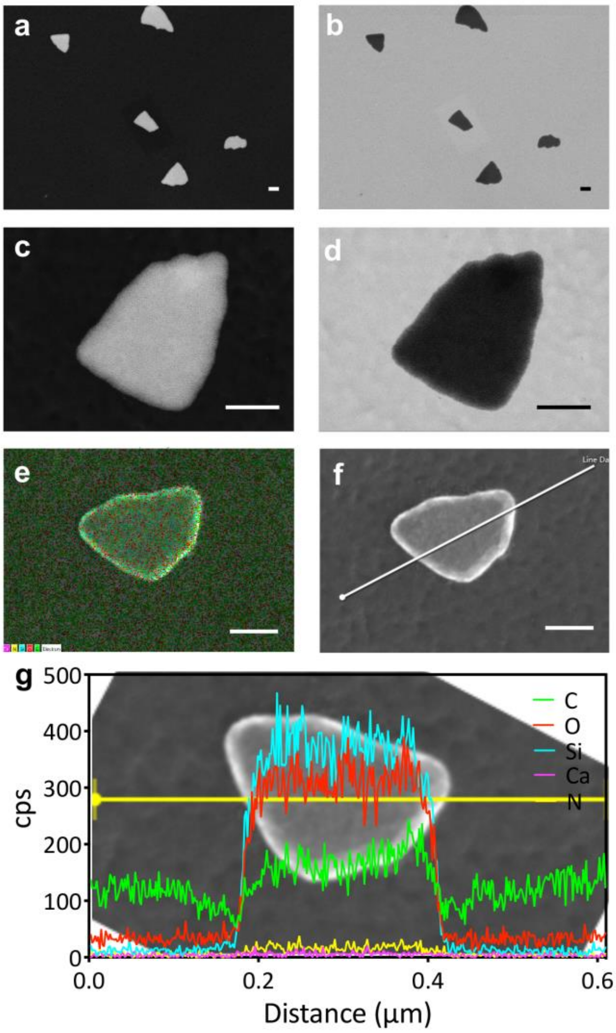

Figure S2. Images of silicene@MSNs and the corresponding elemental mappings of silicene@MSNs loading with ropivacaine. STEM a) dark and b) bright field images at low magnifications and c) dark and d) bright field images of silicene@MSNs at high magnifications. e) STEM and the merged elemental mappings of Si/O/C/N/Ca of silicene@MSNs loading with ropivacaine. f) STEM and g) the corresponding line scanning elemental mappings of Si/O/C/N/Ca of silicene@MSNs loading with ropivacaine. The underneath scale bar: 100 nm.

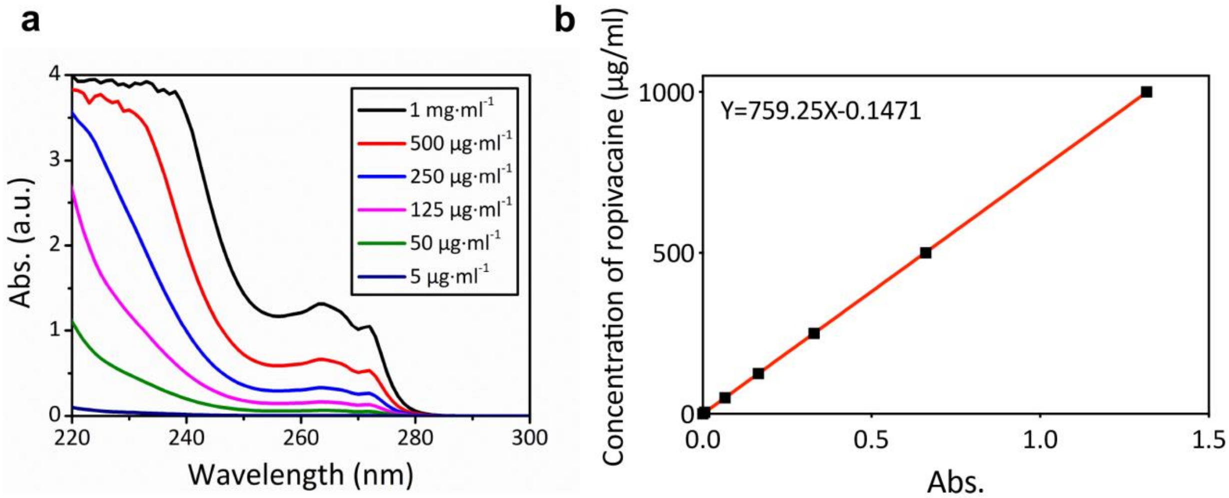

Figure S3. a) UV absorption curve of different concentration of ropivacaine in PBS. Ropivacaine features a characteristic absorption peak at 263 nm. b) The standard curve of the concentration ropivacaine and the UV absorption peak at 263 nm.
